# Operating During the COVID-19 Pandemic: An Emerging Indication for Minilaparotomy Cholecystectomy

**DOI:** 10.7759/cureus.11500

**Published:** 2020-11-16

**Authors:** David M Milne, Johnathan K Jarvis, Rensi E Franklin, Dexter Thomas, Vijay Naraynsingh

**Affiliations:** 1 Surgery, General Hospital Port of Spain, Port of Spain, TTO; 2 Surgery, Medical Associates Hospital, St. Joseph, TTO

**Keywords:** cholecystectomy, covid-19, minilaparotomy, pandemic

## Abstract

COVID-19 has required changes in the practice of surgery to reduce the risk of transmission of the virus. Proposed mitigation strategies include avoidance of aerosol-generating procedures such as laparoscopy. We report two cases where minilaparotomy cholecystectomy was employed to treat benign biliary disease during the pandemic. A review of the literature supports the use of this surgical technique during the COVID-19 pandemic until laparoscopy can be proven to be safe.

## Introduction

The COVID-19 pandemic is the most significant public health crisis faced by all practising doctors in their careers. At the time of writing this article, 37,109,851 confirmed cases had been reported globally with 1,070,355 deaths [1]. This disease has mandated a change in the way we practice surgery.

In order to mitigate the spread of the virus, surgical bodies have been issuing guidance to stratify elective surgeries allowing for the safe postponement of select cases [2,3]. Recommendations also advise caution when undertaking aerosolising procedures such as laparoscopy. While the risk of laparoscopy facilitating COVID-19 spread to health care workers remain theoretical, it should be avoided where possible and only undertaken when the benefits to the patient outweigh the risks to health care professionals [4].

The American College of Surgeons (ACS) has issued guidance on the management of symptomatic gallbladder disease and acute cholecystitis during the coronavirus pandemic [5]. They advise that surgery may be undertaken for severe biliary colic and acute cholecystitis when resources are ample. Laparoscopy has been established as the gold standard for the surgical treatment of benign diseases of the gallbladder. However, ACS guidance does not recommend a specific surgical technique.

Fortunately, minilaparotomy cholecystectomy (MC) employing a 5cm or smaller incision [6] can afford many of the benefits of minimally invasive surgery while forgoing the risks of aerosolisation from the pneumoperitoneum employed in laparoscopy. When compared to laparoscopic cholecystectomy (LC), MC shows no statistically significant difference in mortality and complications. It is also less costly and has a shorter operative time [7].

This report outlines the cases of two patients who presented with complicated gallstone disease during the COVID-19 pandemic. We describe the use of minilaparotomy cholecystectomy in their treatment, as well as review the relevant literature, making a case for this technique to be the standard of care during the COVID-19 pandemic.

## Case presentation

Case 1

B.G. is a 52-year-old female who presented with epigastric pain and vomiting. Over the last four months, she had three prior admissions for gallstone pancreatitis at another institution. Biochemical investigations revealed an amylase of 1813 U/L and a lipase of 2512 U/L. Multiple non-obstructing gallstones were seen on transabdominal ultrasonography, and the common bile duct measured 0.59cm. Overlying bowel gas obscured visualisation of the pancreas. Her Modified Marshall Score was 0, and she was diagnosed with mild acute gallstone pancreatitis [8].

The patient was given opioid analgesia and started on a low-fat diet. Magnetic resonance cholangiopancreatography confirmed the presence of cholelithiasis and ruled out choledocholithiasis. She was subsequently booked for a minilaparotomy cholecystectomy.

Under general anaesthesia, a 5cm transverse subcostal incision was made over the lateral half of the rectus abdominis muscle extending onto the external oblique. The anterior sheath was divided and the rectus muscle preserved by retracting it medially. The posterior sheath was incised and the incision extended on to the internal oblique and the transversus abdominis muscles. A large laparotomy pack was introduced into the wound and placed between the liver and the bowel.

**Figure 1:**
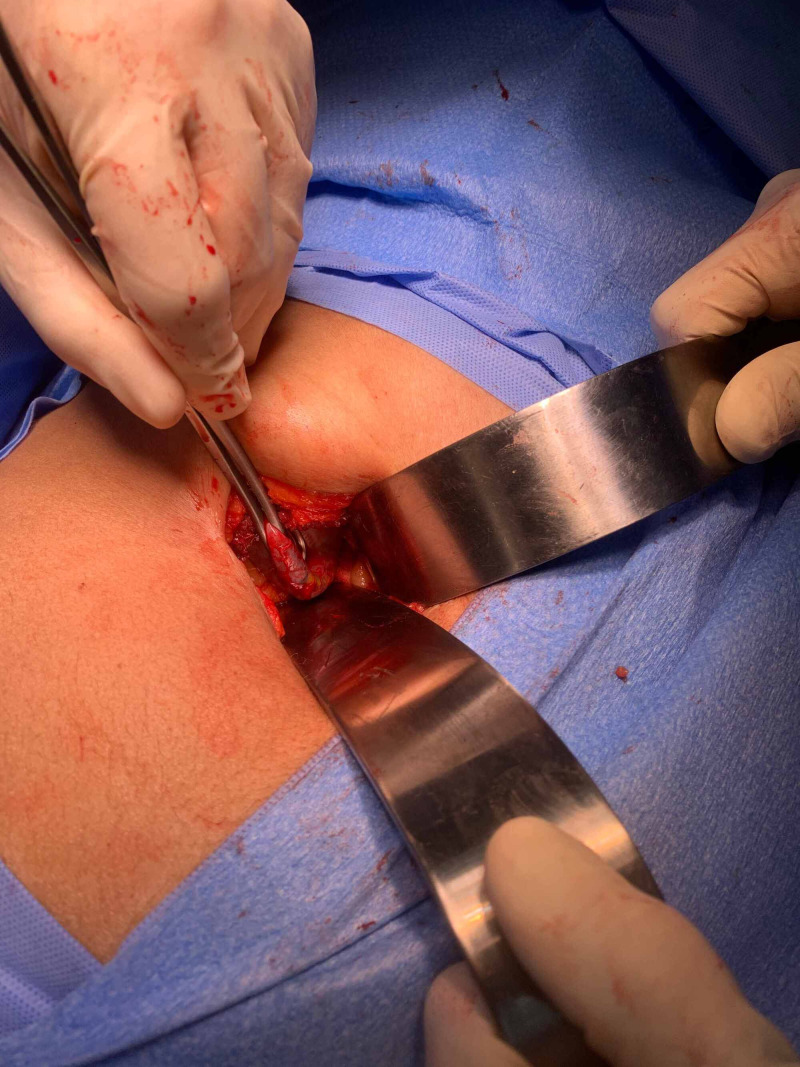
5cm minilaparotomy cholecystectomy incision employing 2 narrow Deaver retractors

Narrow Deaver retractors were used to retract the liver cephalad and the duodenum and adjacent bowel away from the gall bladder (see figure 1). Dissection of the cystic artery and cystic duct were carried out using long conventional instruments. The cystic structures were ligated using 0-silk suture. The gall bladder was dissected, starting from Hartman's pouch advancing towards the fundus (Calot's First fashion) using electrocautery. A local anaesthetic infiltration of 10 mL of 0.25% bupivacaine was injected into the wound before closure in two layers with 0-nylon.

The patient was started on oral diet six hours postoperatively and was ready for discharge 24 hours after surgery. Her postoperative period was uneventful. Histology of the gallbladder confirmed chronic cholecystitis.

Case 2

G.C is a 57-year-old female who presented with classical features of acute cholecystitis. Though moderately obese, there was a palpable, tender mass in the right hypochondrium. She was febrile with a temperature of 38.4 oC and a pulse rate of 96 beats per minute. Her white blood cell count was 16x109/L. Transabdominal ultrasonography showed a distended gall bladder with mural oedema and pericholecystic fluid.

**Figure 2:**
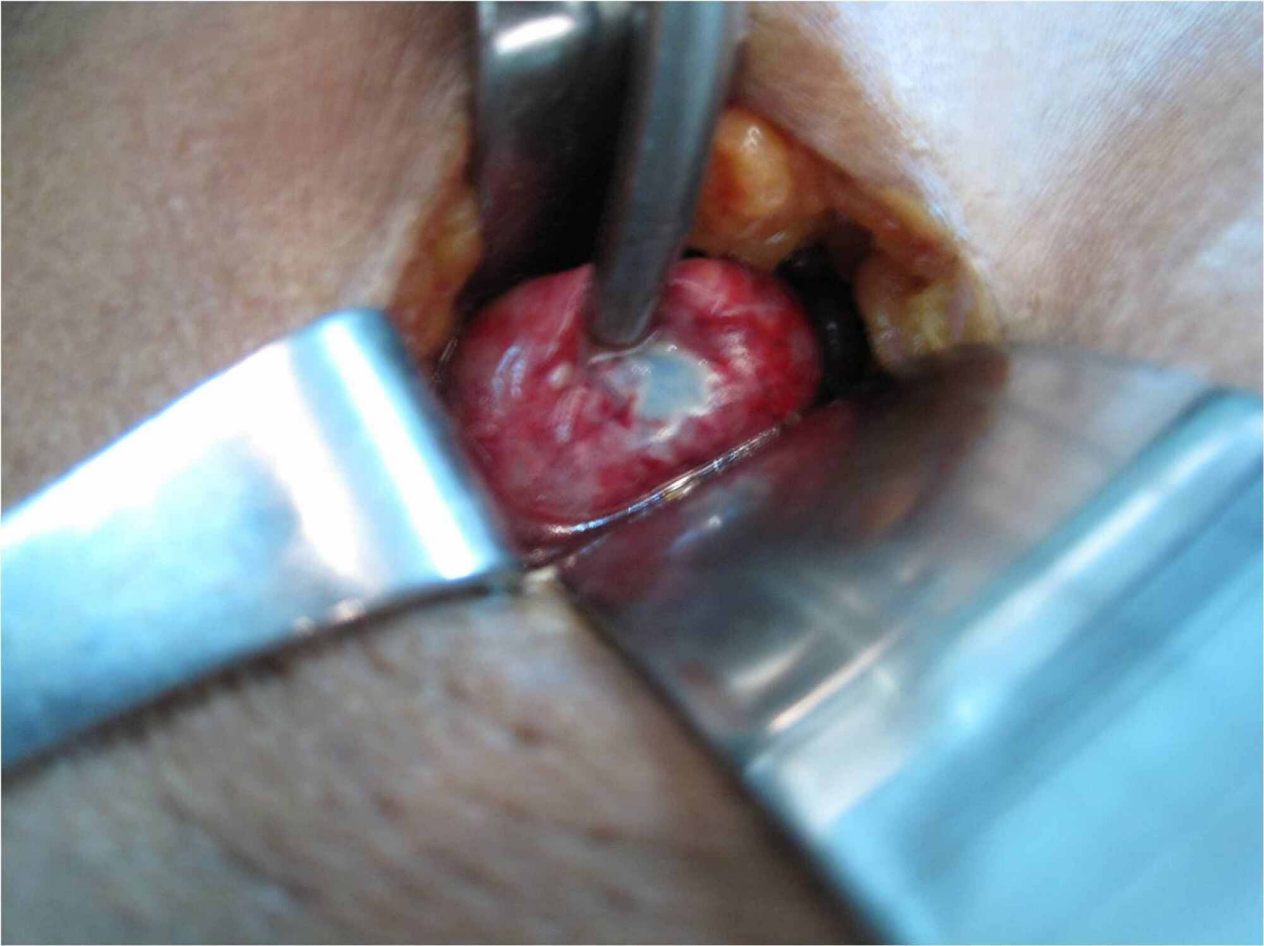
Gangrenous patch on fundus of Gallbladder Empyema seen at minilaparotomy cholecystectomy

**Figure 3:**
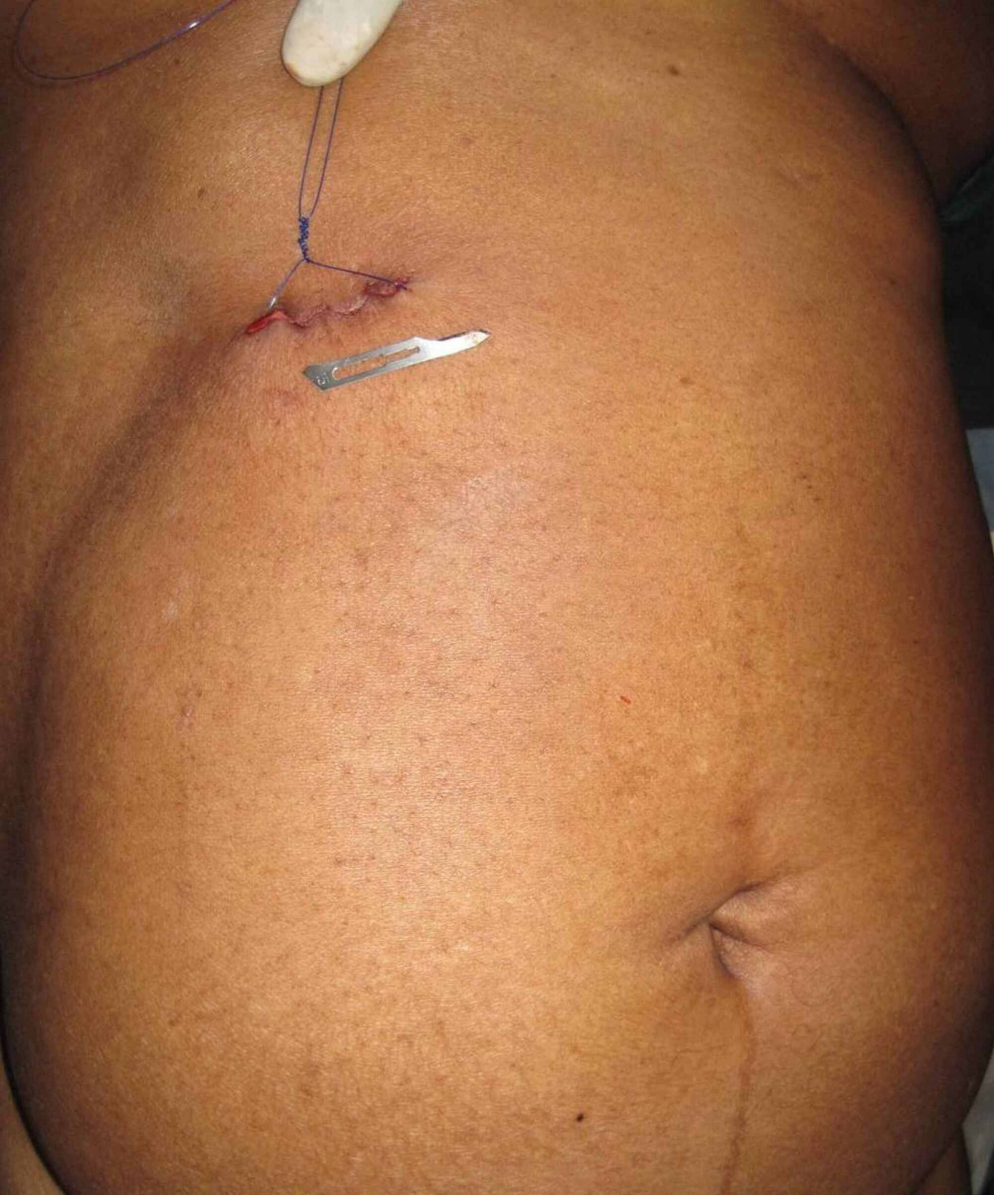
4cm incision seen after closure of minilaparotomy cholecystectomy wound

She was started on intravenous antibiotics. At minilaparotomy cholecystectomy, a mass of adherent omentum was digitally peeled off the gallbladder to reveal a gangrenous patch at the fundus of a gall bladder empyema, shown in figure 2. Routine minilaparotomy cholecystectomy was completed as described in case one utilising a 4cm incision shown in figure 3. She was maintained on intravenous antibiotics and discharged after 48 hours.

## Discussion

Healthcare workers have a 7% greater absolute risk of contracting COVID-19 when compared to the rest of the population [9]. In February 2020, 4.4 % of infections reported in China were in health care workers. Among the 3387 infected, 23 died. Eight (34%) of the deaths were surgeons [10]. In the UK as of April 2020, 18 doctors died, 5 (27%) of whom were surgeons [11].

Our professional dictum: first, do no harm (Primum non nocere) needs to be extended to include both the patient and health care professionals [12]. Strategies must be put in place to reduce the risk of transmission in the hospital. One such strategy is avoiding unnecessary aerosol-generating procedures where ever possible; this includes laparoscopy.

The presence of hepatitis B virus has previously been detected using a polymerase chain reaction test on smoke collected at laparoscopy [13]. Aerosolisation of coronavirus during laparoscopy is yet to be demonstrated, and the risk to health care professionals remains unproven. However, it has been demonstrated that surgeons are exposed to significantly more concentrated aerosolised particles during laparoscopy when compared to open surgery [14]. Until better evidence is available, a cautious approach is suggested.

For benign gallbladder disease, minilaparotomy cholecystectomy (MC) provides an excellent alternative to laparoscopic cholecystectomy (LC). When compared to LC, MC is cheaper [7,15] and faster [7][16]. It is safe in the obese [16] and as an emergency procedure [17]. Research has shown that LC and MC have similar outcomes when looking at mortality [7], complications [7], cosmesis [18], quality of life [15] and same-day surgery rates [19]. When compared to MC, LC offers limited or no advantages to the patient. Therefore it is not justifiable to accept the potential risks associated with laparoscopy. We recommend that all patients requiring surgery for benign biliary disease during the COVID-19 pandemic should be treated with MC once appropriate expertise is available. This recommendation is particularly relevant in low resource settings, which may not have easy access to pre-operative COVID-19 testing, personal protective equipment, or smoke evacuation systems.

MC is performed using simple alterations to the standard technique for open cholecystectomy, making it easy to teach to surgeons and trainees [20]. Furthermore, it provides the opportunity to afford residents much-needed experience in open biliary surgery. While standard equipment is all that is required to perform MC, it may be advantageous to use loupe magnification with headlights for better visualisation and clips to secure the cystic artery and duct given the tight operative field [6].

## Conclusions

Laparoscopy poses a potential risk to health care professionals as a route of transmission of COVID-19. Minilaparotomy cholecystectomy avoids this potential risk while affording many of the benefits of minimally invasive surgery. Further research is needed to quantify the risk of COVID-19 transmission to health care professionals during laparoscopy and other aerosol-generating procedures. Until evidence is available to prove the safety of laparoscopy during the COVID-19 pandemic, minilaparotomy cholecystectomy should be considered as the preferred option to treat benign biliary disease.
